# Refinement of the zebrafish embryo developmental toxicity assay

**DOI:** 10.1016/j.mex.2020.101087

**Published:** 2020-10-07

**Authors:** Jente Hoyberghs, Chloé Bars, Casper Pype, Kenn Foubert, Miriam Ayuso Hernando, Chris Van Ginneken, Jonathan Ball, Steven Van Cruchten

**Affiliations:** a University of Antwerp, Wilrijk, Belgium; b Anju Software, Berchem, Belgium; c University of Exeter, Exeter, UK

**Keywords:** Teratogen, Screening, Alternative, Metabolic activation, Skeletal, Bone staining

## Abstract

Several pharmaceutical and chemical companies are using the zebrafish embryo as an alternative to animal testing for early detection of developmental toxicants. Unfortunately, the protocol of this zebrafish embryo assay varies between labs, resulting in discordant data for identical compounds. The assay also has some limitations, such as low biotransformation capacity and fewer morphological endpoints in comparison with the in vivo mammalian developmental toxicity studies. Consequently, there is a need to standardize and further optimize the assay for developmental toxicity testing. We developed a Zebrafish Embryo Developmental Toxicity Assay (ZEDTA) that can be extended with a metabolic activation system and/or skeletal staining to increase its sensitivity. As such, the ZEDTA can be used as a modular system depending on the compound of interest.

• Our protocol is customized with a metabolic activation system for test compounds, using human liver microsomes. This system ensures exposure of zebrafish embryos to metabolites that are relevant for human risk and safety assessment. As human liver microsomes are toxic for the zebrafish embryo, we developed a preincubation system with an ultracentrifugation and subsequent dilution step.
• Additionally, we developed a skeletal staining protocol that can be added to the ZEDTA modular system. Our live Alizarin Red staining method detects several bone structures in 5-day old zebrafish larvae in a consistent manner.

## Graphical abstract

**Figure:**
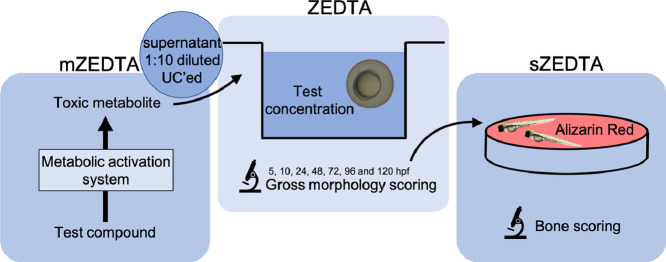

Specifications Table

**Table:** 

Subject Area:	Pharmacology, Toxicology and Pharmaceutical Science
More specific subject area:	Developmental toxicology
Method name:	Zebrafish Embryo Developmental Toxicity Assay (ZEDTA)
Name and reference of original method:	Our method is a modified version of the zebrafish developmental toxicology assay described by Ball et al. in “Fishing for teratogens: a consortium effort for a harmonized developmental toxicology assay. Ball J.S. et al. Toxicol Sci, 2014; 139 (1): p. 210–219″ and the metabolic Danio rerio Teratogenicity (mDarT) test described by Busquet et al. in “Development of a new screening assay to identify proteratogenic substances using zebrafish danio rerio embryo combined with an exogenous mammalian metabolic activation system (mDarT). Busquet F. et al. Toxicol Sci, 2008; 104(1): p. 177–188″.
Resource availability:	*n/a*

## Method details

### Background

**Fig. 1:**
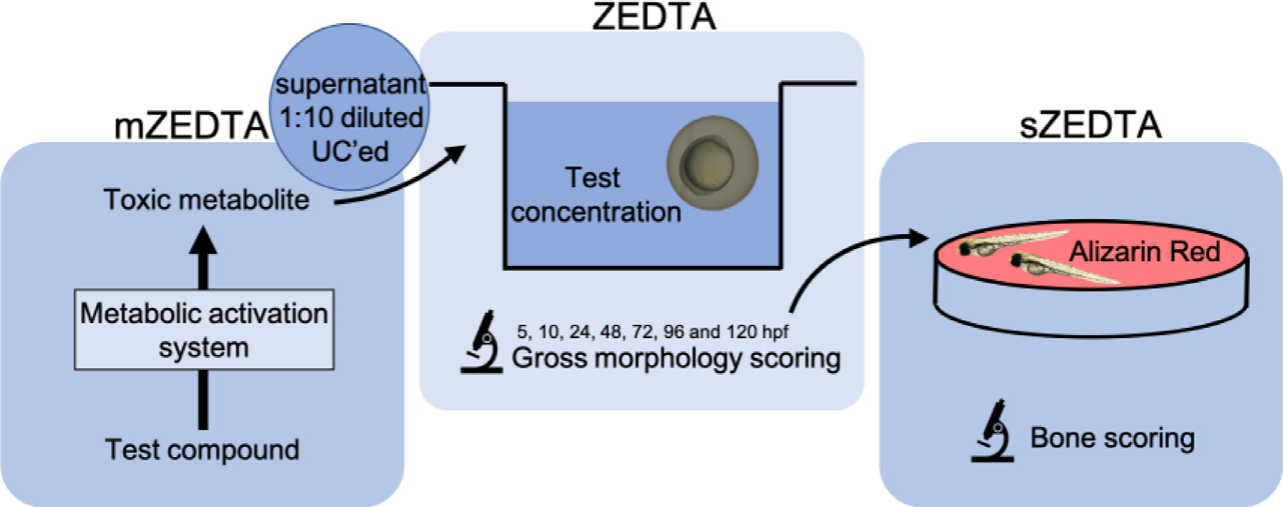
Schematic representation of our ZEDTA, the metabolic (m)ZEDTA and the skeletal (s)ZEDTA. The mZEDTA panel shows the preincubation of the test compound with the metabolic activation system for 1 h. After 1 h, this preincubation mix is ultracentrifuged (UC'ed) at 100,000 x g for 1 h at 4 °C and the supernatant is pipetted and diluted 1:10 to obtain the test solution, as represented in the upper right circle. Zebrafish embryos are then exposed to the test concentration until 120 h post fertilization (hpf) in our ZEDTA protocol (mid panel) and the embryos are scored for gross morphology. For the sZEDTA (depicted in the right panel), the zebrafish embryos are transferred after gross morphology scoring in the ZEDTA to a small petridish with 0.005% Alizarin Red solution. After 1 h the solution is removed, the zebrafish larvae are washed in embryo medium, anesthetized and embedded in agar to be imaged. Finally, the larvae are euthanized and the images are processed and evaluated for bone scoring.

**Fig. 2:**
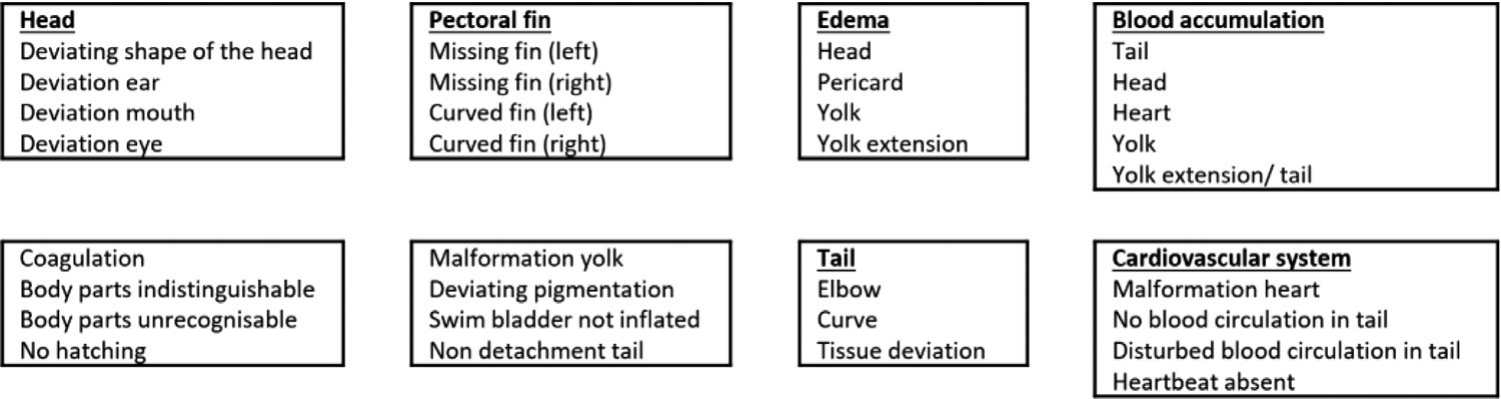
Detailed overview of all morphological endpoints in our ZEDTA.

Developmental toxicity testing mainly relies on in vivo studies in rodent and nonrodent species. As these studies are ethically under discussion, time-consuming and costly, require a lot of test compound and have a low throughput, several pharmaceutical and chemical companies are using *in vitro* and/or in vivo screening assays for early detection of developmental toxicity. One of the alternatives to animal testing that has gained a lot of interest in the last decade is the zebrafish embryo assay. This alternative model has already been validated for assessing acute toxicity of chemicals in view of environmental risk assessment in the so-called (zebra)Fish Embryo acute Toxicity (zFET) test [1]. Several industrial and academic groups also noted the potential of the zebrafish embryo for developmental toxicity testing. Still, the number of morphological endpoints and other factors (such as medium, exposure window, etc.) in this developmental toxicity assay vary between labs, despite harmonization efforts [2,3]. This has led to discordant results for identical compounds. So, there is a clear need for standardization and optimization of this assay. To increase the sensitivity of the assay, we adapted the protocol used by Ball et al. [3] and developed a Zebrafish Embryo Developmental Toxicity Assay (ZEDTA) that can be extended with a metabolic activation and/or skeletal staining protocol (see Table 1, Fig. 1 and procedure section). These additional steps will be further referred to as the metabolic (m)ZEDTA and skeletal (s)ZEDTA, respectively (Fig. 1). The metabolic activation protocol was developed and added to our ZEDTA, because we showed previously that zebrafish embryos have a low biotransformation capacity during a major part of organogenesis and, in contrast to mammals, they cannot rely on the maternal metabolic capacity due to their external development [4,5]. Consequently, test compounds that require bioactivation to exert their toxicity could be missed (and cause false negative results) in a standard ZEDTA. Busquet et al. [7]. were the pioneers in developing a metabolic activation system (MAS) by using rat liver microsomes, but the zebrafish embryos could only be exposed for 1 h (2–3 hpf) to test compounds in this MAS, due to its inherent embryotoxicity. Others tried to expose zebrafish embryos to MAS during the entire period of organogenesis, but they reached only a maximum of 4 h co-incubation with MAS and this only for the older developmental stages [8]. As these groups showed that co-incubation of zebrafish embryos with MAS during the entire exposure period is not feasible, we developed a preincubation system with human liver microsomes as MAS (see mZEDTA procedure below). We used human liver microsomes, as our mZEDTA is aimed for human safety/risk assessment. In addition to the mZEDTA, we developed the sZEDTA in order to extend the number of morphological endpoints in the ZEDTA (Fig. 2 and Table 2). These endpoints are much more limited than in the in vivo mammalian developmental toxicity studies [6] and especially the skeletal endpoints are scarce, as no skeletal staining is performed in a standard ZEDTA. For the skeletal endpoints, we evaluated several skeletal staining methods described in literature (including an Alizarin Red staining protocol for fixed and live larvae and a calcein staining) in collaboration with the University of Exeter. The Alizarin Red staining protocol of live larvae (based on Bensimon-Bristo et al. [9] and personal communications from Dr. C. Hammond (Bristol University, UK) showed the most consistent results (see sZEDTA procedure below). With this staining protocol, we are currently evaluating several proprietary and non-proprietary compounds showing skeletal malformations in rat and/or rabbit as part of a consortium exercise within the European Teratology Society. This evaluation falls out of scope of this methodology paper, which focuses on the staining method and the bones that can be detected in 5-day old zebrafish larvae.

**Table 1:** Comparison between the protocol of the Zebrafish Developmental Toxicology Assay (ZeDTA) by Ball et al. [3] and our Zebrafish Embryo Developmental Toxicity Assay (ZEDTA).

Name of the assay	ZeDTA	ZEDTA
Strain	Several wild type strains	**Wild type AB**
Selection and number of eggs	Select fertilized eggs undergoing cleavage and no signs of irregularities	Select fertilized eggs undergoing cleavage and no signs of irregularities
	12 embryos per concentration, 2 replicates	**20** embryos per concentration, 2 replicates
Temperature	28 °C ± 1 °C	28.5 °C ± 0.2 °C
Chorion	Intact	Intact
Test chamber	24 well-plate (one embryo per well)	**48 well-plate** (one embryo per well)
	1000 µl per well	**500–1000** **µl per well**
Exposure length	Start at gastrulation (4–6 hpf)	Start at gastrulation (from 5.25 hpf)
	Ends at 120 hpf	Ends at 120 hpf
Choice of concentrations	Highest concentration 1000 µM or 100 µM – lowest concentration 0.1 µM	Based on a range finding test with different concentrations
Number of concentrations	4–5 concentrations	By default 3 concentrations, can be reduced or extended
Exposure method	Static	Static, unless nominal chemical concentration < 20% at end of test
Use of solvent	Survival of solvent control should be >= 90% at 120 hpf	Survival of solvent control should be >= 90% at 120 hpf
Morphology scoring system	Morphological scoring based upon Panzica-Kelly et al. [10]	Extended morphological scoring
Medium	0.3x Danieau‘s solution	**TRIS-buffered medium**
pH	7.1–7.3 ± 0.2	7.4 ± 0.2
Conductivity	Not defined	**490–510 µS/cm**
Internal concentrations	Yes	Yes

**Table 2:** General overview of morphological scoring of zebrafish embryos at different developmental stages in our ZEDTA.

	Stage (hpf)						
	5.25	10	24	48	72	96	120
Coagulation	+	+	+	+	+	+	+
Hatching				+	+	+	+
Tail deviation			+	+	+	+	+
Edema			+	+	+	+	+
Blood accumulation			+	+	+	+	+
Malformation of the cardiovascular system			+	+	+	+	+
Malformation of the head			+	+	+	+	+
Malformation of the pectoral fins					+	+	+

hpf = hours post fertilization.

### Procedure

#### ZEDTA

Assess stability and uptake of the test compound (by the zebrafish embryos). Outsource this analysis or perform internally depending on the availability of bioanalytical tools and/or expertise.

Perform the following steps first:

1. For stability assessment, prepare test concentrations in embryo medium (see step 14 in the ZEDTA protocol) and incubate at 28.5 °C ± 0.2 °C.
2. Take samples for analysis at 0 h, 24 h and 120 h and send to the bioanalytical lab. The required volume and dilution steps for sample analysis depend on the bioanalytical method established by the lab.
3. When the test solution is already degraded at 24 h with more than 20% of the nominal concentration (i.e. concentration at 0 h), the test compound is too unstable in the embryo medium. Do not proceed further.
4. When the test solution is degraded at 120 h with more than 20% of the nominal concentration, use a semi-static exposure approach in step 15 of the ZEDTA protocol, i.e. renew your test solutions every 24 h.
5. For assessment of uptake, i.e. the concentration of the test compound in zebrafish embryos, sample zebrafish embryos (see steps 1–11 of the ZEDTA protocol for embryo collection) that have been exposed to the test concentrations (from 5.25 hpf onwards) at 24 hpf and at 120 hpf. The required number of embryos depends on the established bioanalytical method.
6. Use the washing protocol and further preparatory steps provided by the bioanalytical lab.
7. Analysis and reporting of the internal concentrations by the bioanalytical lab.
8. When the analytical data show no uptake over the exposure period (at 24 hpf and 120 hpf), do not proceed further.
9. When lower or higher internal concentrations of the test compound are noted at 120 hpf than at 24 hpf and this cannot be explained by the stability data, the test compound was metabolized by or accumulated in the zebrafish embryos, respectively, and this has to be considered when interpreting the data in step 16 of our ZEDTA protocol.
10. Proceed to the ZEDTA protocol below.

Follow these steps of our ZEDTA protocol:

1. Use adult zebrafish breeding stock (*Danio rerio*, AB zebrafish line, GIGA, University of Liège) kept in tanks of approximately 50 l
2. Set water temperature to 28.5 °C ± 0.2 °C; conductivity 500 ± 25 µS/cm (adjusted with Instant Ocean sea salts), pH 7.5 ± 0.3 (adjusted with NaHCO3).
3. Renew water when ammonia, nitrite and nitrate reach detectable levels.
4. Apply a light cycle with 14/10 h light/dark.
5. Feed fish daily with thawed food (*Artemia nauplii, Daphnia, Chironomidae* larvae or *Chaoborus* larvae) twice a day, as well as granulated food at a rate of 2% of their mean wet weight per feeding, twice a day.
6. Put the fish in a net in the tank the day before mating to avoid eating of the eggs.
7. The following morning, allow fish to spawn eggs and fertilize them for about 45 min.
8. Collect fertilized eggs from the bottom of the tank by siphoning them out with a tube.
9. Remove feces and coagulated eggs and rinse remaining embryos in TRIS buffered medium, *i.e*. 0.294 g CaCl_2._H_2_O, 0.123 g MgSO_4_•7H_2_O, 0.059 g NaHCO_3_, 0.005 g KCl and 0.1 M tris-HCl (pH 7.5) dissolved in 1 l reverse osmosis water (further referred to as embryo medium).
10. Check embryos under a stereomicroscope for normal cell division within 2 hpf.
11. Discard unfertilized eggs or asymmetric embryos and replace them with healthy eggs until sufficient embryos are obtained (see step 14).
12. Transfer all selected embryos randomly into a 48-well plate (one embryo per well), filled with min. 500 µl embryo medium per well.
13. The experiment is valid when the fertilization rate is >90% and mortality of the controls is <10% at the end of the experiment.
14. Use 20 embryos per replicate (2 replicates, performed in different weeks) of each control group (medium and/or solvent) and each test group. Classically 3 concentrations (Low, Mid, High) of the test compound are used in order to determine a NOAEL (non-observed adverse effect level), but the number of test groups can be reduced (e.g. for back-up compounds) or extended with more concentrations of the test compound (and its human metabolites after preincubation in a metabolic activation system, when applicable (see *mZEDTA*)). To establish concentration-response curves, 5 or more concentrations of the test compounds may be needed.
15. Rear medium control embryos in embryo medium until 120 hpf. Transfer solvent controls and test groups at 5.25 hpf from embryo medium into the solvent concentration or test solution, respectively, and they remain in the solvent/test solution until 120 hpf. When using solvents other than 0.1% DMSO, perform a solvent control experiment first, as the solvent (concentration) may be toxic for zebrafish embryos.
16. Evaluate the zebrafish at 5.25, 10, 24, 48, 72, 96 and 120 hpf for the morphological parameters depicted in Table 2. Use the earliest timepoint (5.25 hpf) to monitor embryo quality at the start of exposure. Larvae at 96 and 120 hpf must be evaluated under anesthesia with MS-222 (0.2 g/l in embryo medium) due to their ability to swim, which hampers the morphological evaluation. Use supplementary Figs. 1–8 for the binary classification of the gross morphology endpoints. Use score 0 for normal morphology and score 1 for abnormal morphology. Fig. 3 depicts some of the most common malformations in zebrafish at 96 hpf. The percentage of malformed embryos is calculated by dividing the number of alive larvae having one or more malformations with the total number of living larvae, multiplied by 100.

**Fig. 3:**
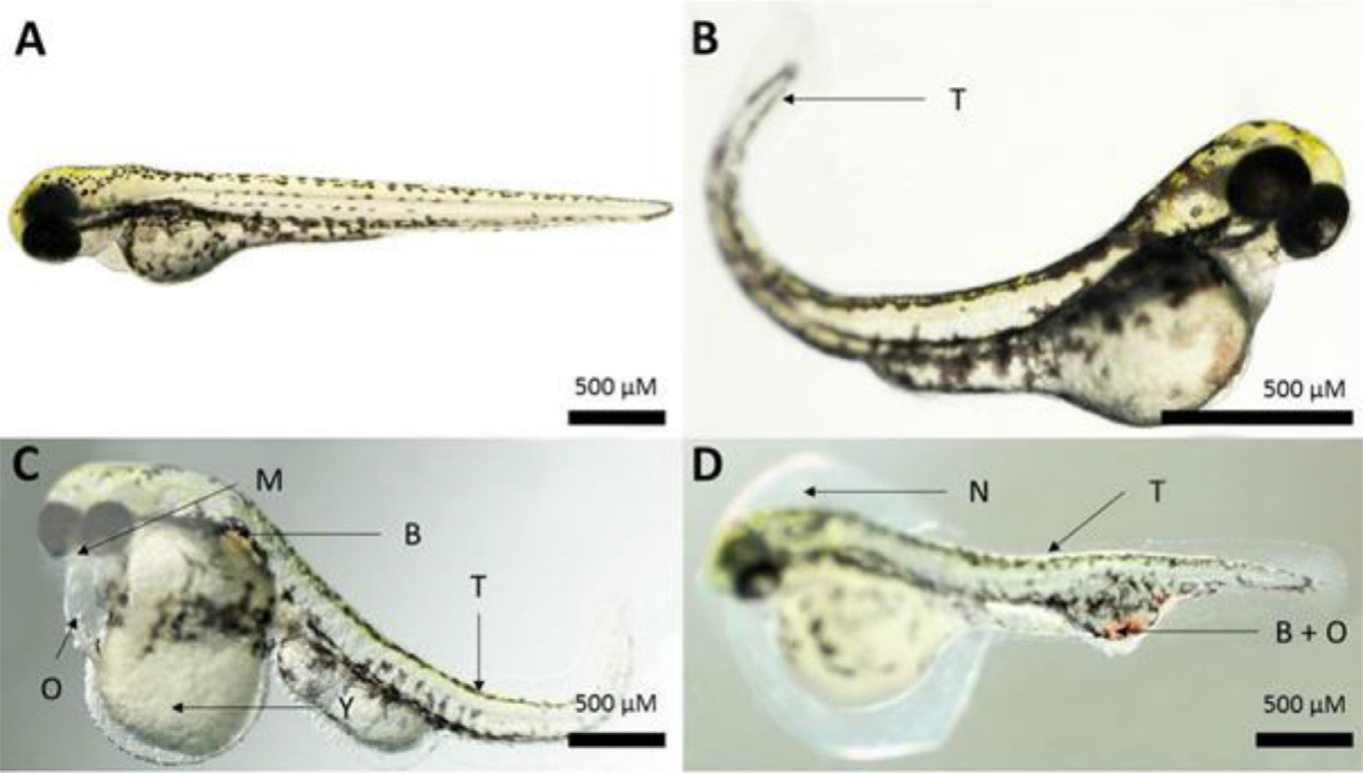
Overview of several malformations that can be observed in 96 hpf zebrafish larvae. A. Normal zebrafish larva. B. Tail malformation (curve) (T). C-D. Several malformations are present. Abbreviations: B: blood accumulation; M: malformation mouth (underdeveloped); N: non-hatching; O: Edema; T: tail malformation (curve); Y: malformation yolk. Adapted from [11].
17. Euthanize the larvae by means of an overdose of MS-222 (1 g/l in embryo medium) after the last gross morphology scoring, Alternatively, use hypothermic shock (in e.g. ice water) for at least 12 h.

#### Metabolic (m)ZEDTA

Follow steps 1 to 13 of our ZEDTA protocol and then continue with the following steps:

14. Preincubate the test compound (one or more concentrations) with human liver microsomes (pooled, 50 donors, Thermo Fischer scientific, USA) at 200 µg/ml and NADPH tetrasodium salt (1.25 mM) in embryo medium for 1 h at 28.5 °C. In addition to the test suspension(s), include a medium control and a blank control, i.e. the preincubation mix without test compound.
14′. After 1 h preincubation, the test suspension and blank control are ultracentrifuged at 100 000 x g for 1 h at 4 °C.
14″. Pipet the supernatant of the blank control and the test suspension, containing the test compound and its metabolites, and dilute the supernatant 1:10 in embryo medium.
15–17. Follow the remaining steps in our ZEDTA. In case of negative results, proceed to step 18.
18. Increase the concentration of the test compound (up to substrate inhibition) in step 14 and follow the remaining steps in the mZEDTA. In case of negative results, proceed to step 19.
19. Determine the metabolite concentrations in the test solution of step 18. In case of low metabolite concentrations (in most cases < µM range), concentrate the test solution. Different procedures can be applied. Discuss with a (bio)analytical expert for your compound(s) of interest.

#### Skeletal (s)ZEDTA

**Fig. 4:**
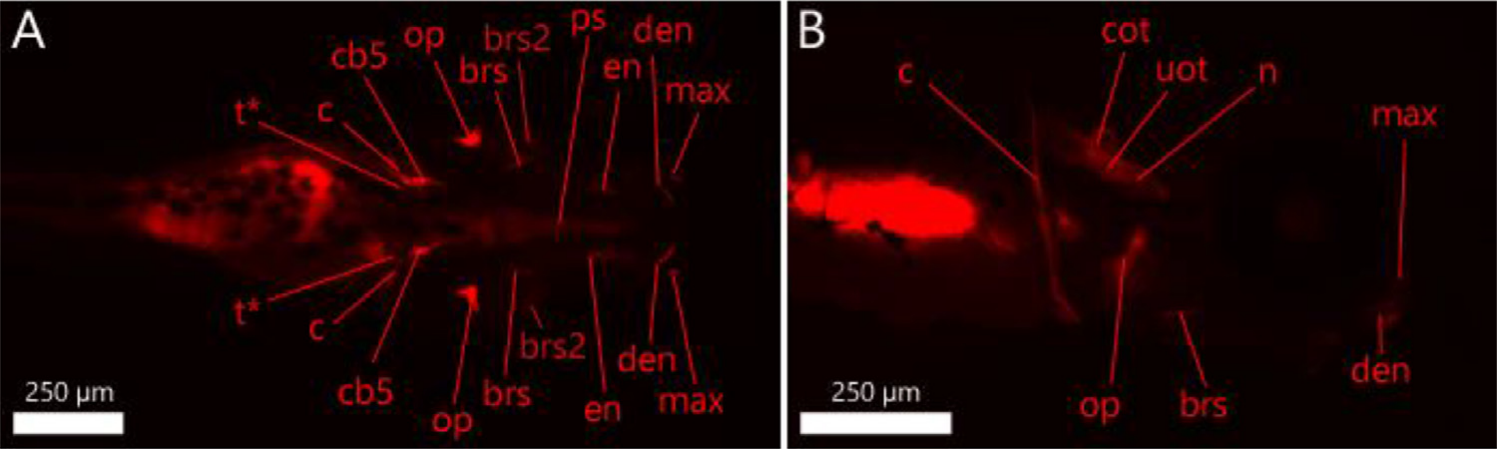
Zebrafish larvae at 120 hpf with bone structures that are stained with 0.005% Alizarin Red. Left panel (A) shows a dorsal view. Right panel (B) shows a lateral view. The abbreviations are depicted in Table 3. * t should be present at 120 hpf, but cannot be distinguished from cb5.

**Fig. 5:**
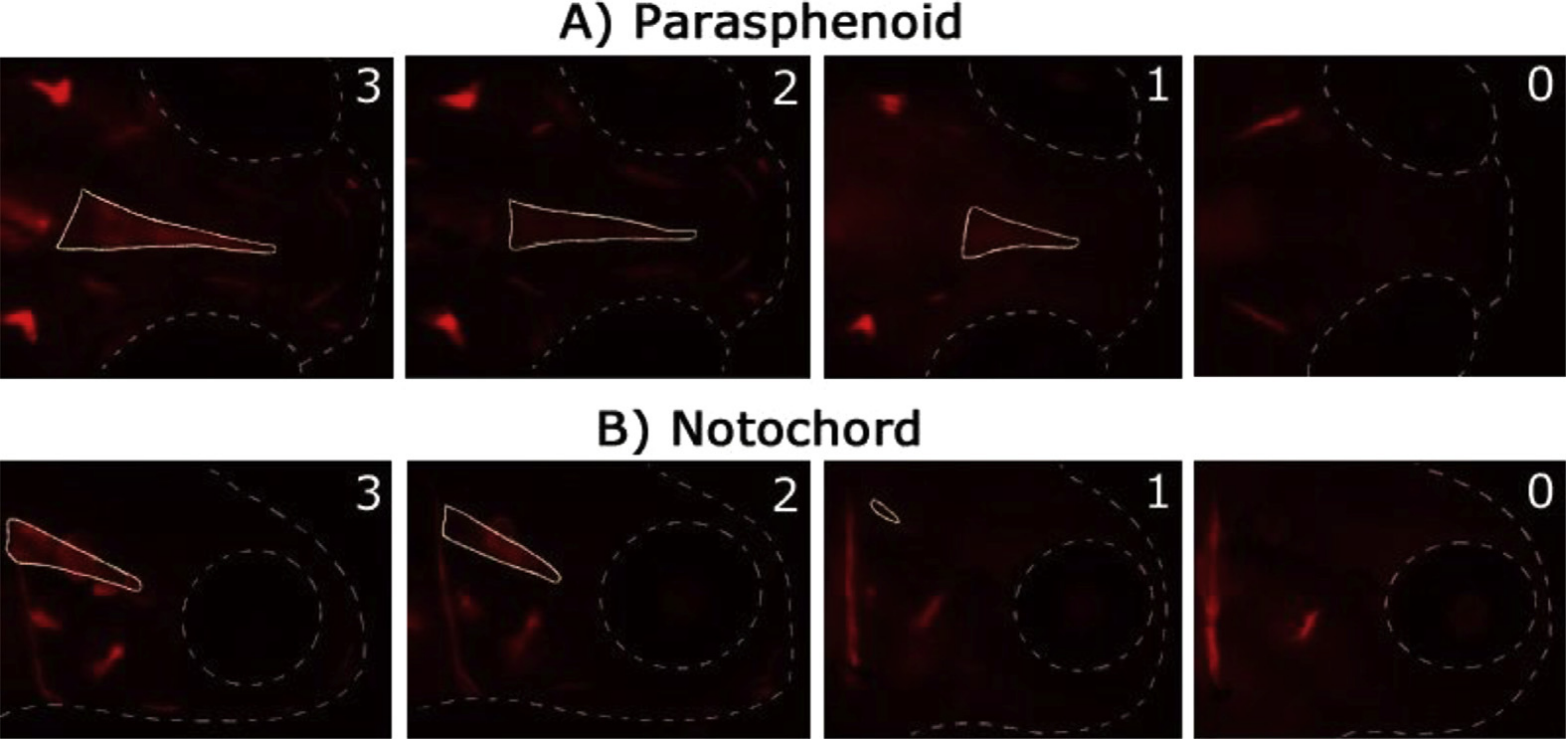
Illustration of the intensity scoring system for 2 bones at 120 hpf (0 = not stained; 1 = weakly stained; 2 = moderately stained; 3 = heavily stained). The upper panel (A) is a dorsal view, the lower panel (B) is a lateral view. The stained bone of interest is delineated with a full line. The head and eye are delineated with a dotted line.

Follow steps 1 to 16 of our ZEDTA protocol and then continue with the following steps:

16′. Transfer the larvae at 120 hpf into a small petridish and remove the solution.
16″. Add 10 ml 0.005% Alizarin Red solution to the petridish.
16″′. Remove the solution after 1 h and wash the larvae in embryo medium.
17. Transfer the larvae in MS-222 (0.2 g/l in embryo medium) until they lose their dorsal-ventral balance.
18. Embed the larvae in 1% low gelling temperature agar containing 0.2 g/l MS-222 and image them with an Olympus SZX16 (6.3x mag) scope (Olympus, UK) in lateral and dorsoventral position with Prior 200 Lumen illumination (100%) with RFP at 620 nm for 100 ms capture in µManager (v1.4) with 8 images per stack (1 stack per position and thus 16 images in total) at 3 second intervals using a Zyla 4.2 sCMOS camera (Andor, UK).
19. Euthanize the larvae by means of an overdose of MS-222 (1 g/l in embryo medium). Alternatively, use hypothermic shock (in e.g. ice water) for at least 12 h.
20. Process the images in ImageJ and evaluate the intensity and shape of the bone structures depicted in Table 3 and Fig. 4. For intensity, use score 0 when the structure is not stained, score 1 when the structure is weakly stained, score 2 when the structure is moderately stained and score 3 when the structure is heavily stained (see Fig. 5). Select for each bone structure of each larva in each group, the image with the highest intensity score to determine the final score of the bone. For shape, use score 0 when the structure is normal and use score 1 when malformed.

**Table 3:** Bone structures in zebrafish at 120 hpf.

Structure (+ abbreviation)	120 hpf
entopterygoid (en)	+
operculum (op)	+
parasphenoid (ps)	+
cleithrum (c)	+
notochord (n)	+
ceratobranchial V (cb5)	+
pharyngeal teeth (t)	+
utricular otoliths (uot)	+
circle saccular otoliths (cot)	+
branchiostegal rays (brs and brs2)	+
dentary (den)	/
maxilla (max)	/

+ stained at 120 hpf, / can be stained as the structure starts to develop around 120 hpf.

## Method validation

### ZEDTA

In order to validate our ZEDTA protocol, we used 30 mM trimethadione (Tebu-bio, Boechout, Belgium) in embryo medium as test solution. Trimethadione is an anticonvulsant and human teratogen. We used one concentration in our ZEDTA, as Weigt et al. [12] already showed that exposure of zebrafish embryos to 20 mM and 40 mM trimethadione from 2 hpf until 72 hpf caused 33% and 88% malformed embryos, respectively. Our experiment was valid, as the medium controls had a coagulation of less than 10% (see Table 4) and the fertilization rate was above 90% (data not shown). The statistical analysis was carried out with GraphPad Prism 7 (San Diego, CA, USA). Morphological data were analyzed by means of the Fisher's exact test. Results were considered significant at *P* < 0.05. After comparing the morphological effects in the replicates, no replicate showed more or less malformations than the other for any of the parameters (*P* > 0.05). Therefore, the data of the replicates were pooled. The results are depicted in Table 4. 30 mM trimethadione clearly showed malformations at the end of the exposure period with several organs affected. The higher than expected percentage of affected embryos (93%) with 30 mM trimethadione in our experiment compared to the study by Weigt et al. [12] can be explained by the longer exposure period in our ZEDTA, as the last morphological scoring in [12] was done at 72 hpf. At 72 hpf, we obtained 60% of malformed embryos in our ZEDTA and several morphological endpoints were not affected yet (Table 5). These findings emphasize the importance of the exposure duration (i.e. the entire period of organogenesis) and not only the dose/concentration of the developmental toxicant.

**Table 4:** Overview of coagulation and malformations in the group exposed to 30 mM trimethadione at the end of the experiment.

Affected parameter	Medium control	30 mM trimethadione
Coagulation	8%	0%⁎⁎⁎
**Malformed embryos**	**2%**	**93%**⁎⁎⁎
No hatching	0%	52%⁎⁎⁎⁎
Curved tail	2%	60%⁎⁎⁎
Edema pericard	2%	75%⁎⁎⁎
Blood accumulation yolk	0%	12%*
Malformation yolk	2%	33%⁎⁎⁎
No blood circulation	2%	50%⁎⁎⁎
Absence of a heartbeat	2%	13%*
Deviating shape of mouth	2%	22%⁎⁎

⁎ *P* < 0.05.

⁎⁎ *P* < 0.01.

⁎⁎⁎ *P* < 0.0001.

**Table 5:** Overview of coagulation and malformations in the group exposed to 30 mM trimethadione at 72 hpf.

Affected parameter	Medium control	30 mM trimethadione
Coagulation	8%	0%
**Malformed embryos**	**2%**	**60%**⁎⁎⁎
No hatching	38%	80%*
Curved tail	4%	42%⁎⁎
Edema pericard	2%	55%⁎⁎⁎
Blood accumulation yolk	0%	5%
Malformation yolk	2%	10%
No blood circulation	2%	18%⁎⁎
Absence of a heartbeat	0%	3%
Deviating shape of mouth	2%	0%

⁎ *P* < 0.05.

⁎⁎ *P* < 0.01.

⁎⁎⁎ *P* < 0.0001.

### mZEDTA

For the mZEDTA, we performed morphological experiments with undiluted and 1:10, 1:20 and 1:30 diluted supernatant of an ultracentrifuged blank control (i.e. preincubation mix of human liver microsomes at 200 µg/ml and NADPH tetrasodium salt (1.25 mM) in embryo medium). Our experiment was valid, as the medium controls had a coagulation of less than 10% (see Table 6) and the fertilization rate was above 90% (data not shown). The morphological data analysis was carried out as described above in the ZEDTA experiment with 30 mM trimethadione. The results are depicted in Table 6. When using the undiluted supernatant of the blank control, a slight increase in yolk malformations was noted (*P* < 0.01), although the total number of affected embryos did not reach statistical significance (*P* = 0.0528). Exposing zebrafish embryos to the 1:10 or higher diluted supernatant of the ultracentrifuged preincubation system showed no effect on any of the morphological parameters. These data confirm that our preincubation protocol is not embryotoxic and consequently zebrafish embryos can be exposed during the entire organogenesis to 1:10 diluted supernatant of our ultracentrifuged metabolic activation system. However, as the 1:10 dilution may also dilute the effect of the toxic metabolite in the supernatant, one must be cautious and the activity of metabolic activation system, i.e. the obtained metabolite concentrations, must be assessed, especially in case of negative results.

**Table 6:** Overview of coagulation and malformations in the group exposed to the blank control after ultracentrifugation.

Affected parameter	Medium control	Ultracentrifuged blank control
Coagulation	5%	10%
**Malformed embryos**	3%	17%
Edema pericard	3%	3%
Edema yolk	0%	3%
Blood accumulation yolk	0%	3%
Malformation yolk	0%	17%⁎⁎
Malformation heart	0%	3%
No blood circulation	0%	3%
Deviating shape of head	0%	3%
Deviating shape of mouth	0%	3%

⁎⁎ *P* < 0.01.

To test this part of our protocol, we used carbamazepine as tool compound. Carbamazepine is an anti-epileptic drug and a human teratogen. It is believed that its metabolite carbamazepine-10–11-epoxide is causing the malformations. As depicted in Table 7, we used 4 conditions, i.e. 250 µM carbamazepine (Sigma-Aldrich, Diegem, Belgium) and 125 µM carbamazepine-10–11-epoxide (Sigma-Aldrich, Diegem, Belgium) as internal controls for our analytical measurement, undiluted supernatant after step 14′ to assess consumption of carbamazepine and our test solution, 1:10 diluted supernatant after ultracentrifugation of the preincubation mix, to determine the actual concentration of the metabolite carbamazepine-10–11-epoxide in our mZEDTA. Prior to the analysis of the test conditions, we determined the stability of 500 µM carbamazepine in embryo medium (according to the stability protocol in our ZEDTA). Uptake by the zebrafish embryos was not assessed, as a dose-response in morphological defects had already been established by others for this compound [12].

**Table 7:** Identification of the samples that were analyzed with LC-MS for presence of carbamazepine and carbamazepine-10,11-epoxide.

Sample Content	Estimated molar concentration (µM)	Estimated mass concentration (µg/ml)
Parent (Carbamazepine)	250 µM	59.0 µg/ml
Ultracentrifuged (UC'ed) preincubation mixture (Carbamazepine)	250 µM	59.0 µg/ml
1/10 Ultracentrifuged (UC'ed) preincubation mixture (Carbamazepine)	25 µM	5.90 µg/ml
Human metabolite (Carbamazepine-10,11-epoxide)	125 µM	31.5 µg/ml

The following analytical protocol was used:

First, the carbamazepine samples (parent and ultracentrifuged preincubation mixture in Table 7) were diluted in water (HPLC grade) by a factor of 10. These diluted samples were once again diluted by a factor of 10 in 70:30 (v/v) water:acetonitrile (HPLC grade) containing 0.39 µM lamotrigine (Sigma-Aldrich, Diegem, Belgium) as an internal standard. In order to quantify the concentrations of carbamazepine and carbamazepine-10,11-epoxide in these samples, standard curves (in replicate) for carbamazepine (17.5 - 50,000 ng/mL) and carbamazepine-10,11-epoxide (1.7 – 50,000 ng/mL) were prepared. These samples were subsequently diluted by a factor of 10 in water:acetonitrile containing 0.39 µM lamotrigine. The samples were then stored at −80 °C upon use.

The analytical investigation was realized on an Acquity Ultra Performance LC with sample manager, binary solvent manager, diode array detector (DAD) and a triple quadrupole (TQ) detector (ACQUITY UPLC-TQ detector, Waters, Milford USA), equipped with Masslynx software (version 4.1). Chromatographic separation was performed on an Acquity UPLC HSS T3 (2.1 × 100 mm; 1.8 µm) column (Waters, Milford USA) and elution was conducted with a mobile phase consisting of water with 0.1% formic acid (A) and acetonitrile containing 0.1% of formic acid (B). Chromatographic separation of the three analytes was accomplished in 6 min, using a flow rate of 0.5 ml/min and the solvent gradient program was set as follows: 85% A / 15% B (0–0.5 min); 85–0% A / 15–100% B (0.5–3.3 min); 0% A / 100% B (3.3–4.4 min); 0–85% A / 100–15% B (4.4–4.5 min); 85% A / 15% B (4.5–6 min). The column was set at 40 °C and the injection volume was 10 µl. As mass spectrometric conditions, the following parameters were used for data acquisition in positive ionization mode: capillarity voltage 3.5 kV, extractor voltage 3 V, cone voltage 35 V, Rf lens 0.1 V. The source temperature was set at 120 °C and the desolation temperature was set at 450 °C. The desolvation gas flow was 1000 l/h and the cone gas flow was 50 l/h.

Quantification of the analytes was realized via multiple reaction monitoring in positive ion mode of the ion transitions of carbamazepine, carbamazepine-10,11-epoxide and the internal standard, lamotrigine. Following transitions were chosen as quantifier and qualifiers for carbamazepine *m/z* 237→194 (cone voltage 37 V, Ecollision 22 kV) and *m/z* 237→165(cone voltage 37 V, Ecollision. For carbamazepine-10,11-epoxide the chosen transitions were *m/z* 253 →180 m/z (cone voltage 25 V, Ecollision 38 kV) and *m/z* 253→236 (cone voltage 25 V, Ecollision 12 kV). To finish, for the internal standard lamotrigine, the transitions followed were *m/z* 256→ 211 (cone voltage 25 V, Ecollision 25 kV) and *m/z* 256→108 (cone voltage 56 V, Ecollision 35 kV).

**Fig. 6:**
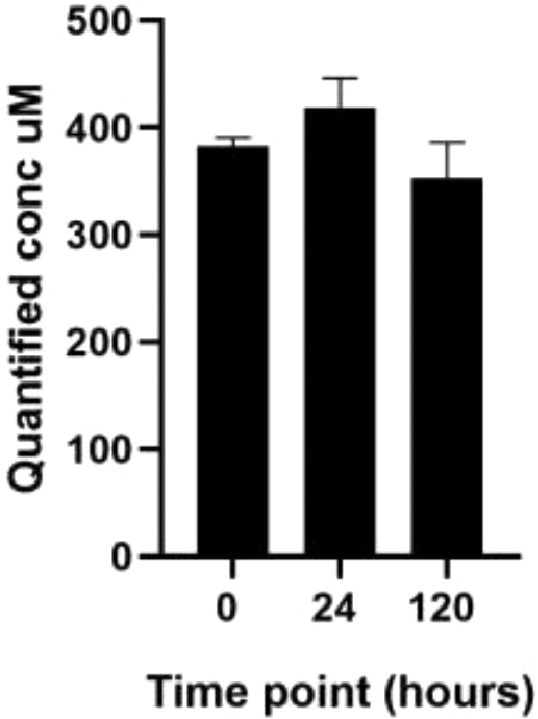
Quantified concentrations of carbamazepine (500 µM, 2 replicates) in embryo medium at 0, 24 and 120 h.

The following results were obtained:

Carbamazepine was stable over the entire period of organogenesis (Fig. 6).

**Fig. 7:**
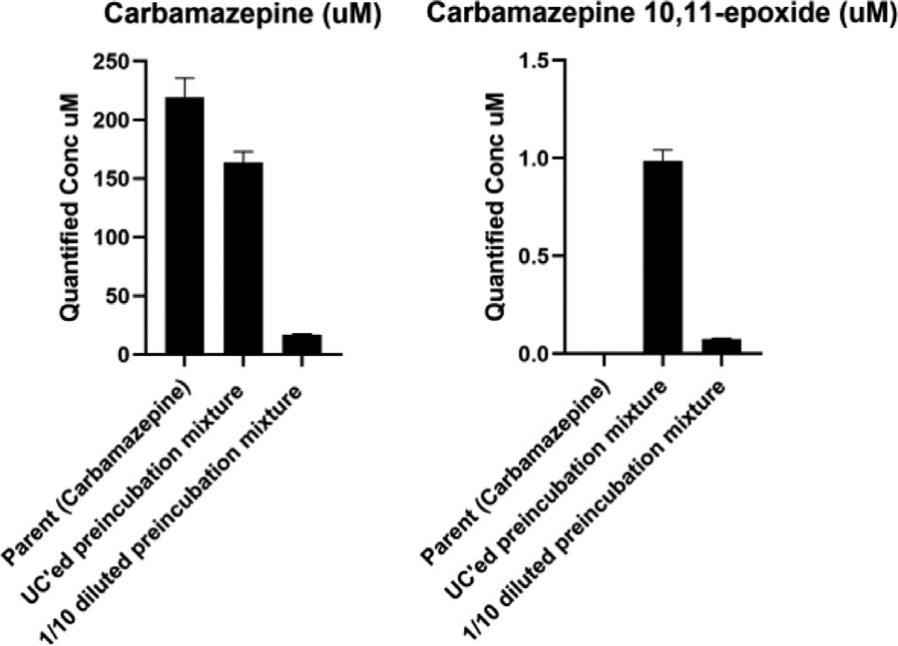
Quantified concentrations (µM (= uM); 2 replicates) of carbamazepine (left panel) and its toxic metabolite carbamazepine 10,11-epoxide (right panel) in the parent compound solution (without incubation), the undiluted supernatant of the ultracentrifuged (UC'ed) preincubation mix and the test solution, i.e. 1:10 diluted supernatant of the ultracentrifuged (UC'ed) preincubation mix.

Carbamazepine was metabolized by 25.3 ± 2.9% after 1 h incubation in our metabolic activation system, indicated by the remaining concentration of carbamazepine in the supernatant of the ultracentrifuged preincubation mixture (Fig. 7). The 1:10 dilution of this supernatant showed about a 10-fold lower carbamazepine concentration. For carbamazepine 10,11-epoxide, no metabolite was found in the control sample of carbamazepine (parent compound without preincubation), as it should be. In the ultracentrifuged and 1:10 ultracentrifuged samples of the preincubation mix, the concentration of carbamazepine 10,11-epoxide was about 150-fold lower than carbamazepine under these conditions, resulting in a concentration of about 0.1 µM carbamazepine 10,11-epoxide in the test solution (see Fig. 7). As most developmental toxicants exert their effect in zebrafish embryos in the µM to mM range, 0.1 µM carbamazepine 10,11-epoxide is believed to be too low to further assess this test solution morphologically in our ZEDTA. We will first perform a range finding study with different concentrations of carbamazepine 10,11-epoxide in order to determine the concentration that causes malformations in our ZEDTA. Depending on this outcome, we will increase the concentrations of carbamazepine in our metabolic activation system (up to substrate inhibition) first (as depicted in step 18 of our mZEDTA protocol) and when the ZEDTA remains negative, we will concentrate our test solution (see step 19 of the mZEDTA) by using a volatile solvent, such as methanol, followed by evaporation when higher concentrations of carbamazepine 10,11-epoxide are needed. This latter procedure has recently been proven successful for chemicals when using zebrafish embryos/larvae of 72 hpf [13].

So, in conclusion we developed a non-embryotoxic metabolic activation protocol that is generating the toxic metabolite. When necessary, the metabolite concentrations can be increased.

### sZEDTA

**Fig. 8:**
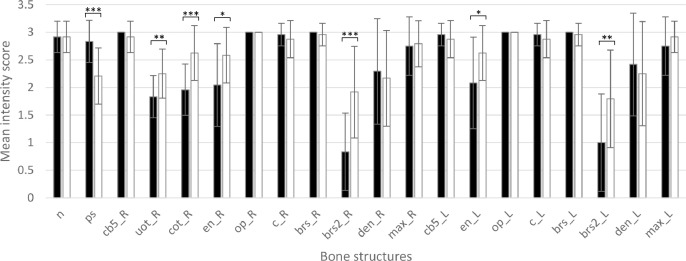
Mean intensity score (and SD) of each bone structure in 120 hpf zebrafish larvae (*n* = 20/group) for the solvent control group (DMSO; black bars) and for the group exposed to 12.5 µM rosiglitazone (white bars). For abbreviations of the bone structures, see Table 3. L: Left; R: right; * *P*<0.05; **P<0.01; ***P<0.0001; t_L, t_R, uot_L and cot_L could not be scored because they were hidden behind other bone structures.

**Fig. 9:**
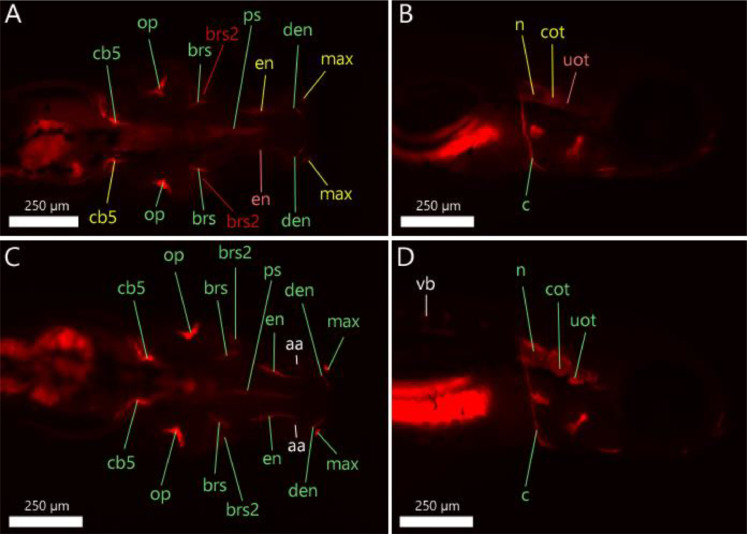
Solvent control (A-B) and 12.5 µM rosiglitazone exposed (C-D) zebrafish larvae at 120 hpf with bone structures that are stained with 0.005% Alizarin Red. Left panels (A-C) show a dorsal view. Right panels (B-D) show lateral view. The abbreviations are depicted in Table 3. The different colors of the abbreviated bone structures indicate different scores of intensities (green = score 3; yellow = score 2; pink = score 1; red = score 0). White-indicated structures (aa= anguloarticular bone; vb = vertebra) are structures that were stained, but expected at a later developmental stage.

For the skeletal staining protocol, we used rosiglitazone (Merck Life Science UK Ltd, Dorset, UK) as test compound. Rosiglitazone is an anti-diabetic compound (PPAR gamma agonist) shown to inhibit osteoblast differentiation and activate osteoclast differentiation in several human in vitro and transgenic mice models. As data were lacking for the zebrafish embryo, we first performed a dose range finding study in order to find non-lethal concentrations of rosiglitazone. Concentrations of 15 µM and 20 µM caused lethality in zebrafish embryos, whereas 12.5 µM did not show any effects on survival nor gross morphology (data not shown). Therefore, a test solution of 12.5 µM rosiglitazone in 0.1% DMSO embryo medium was chosen for our sZEDTA protocol. A medium and solvent control were included. Statistical analysis was carried out with GraphPad Prism 8 (San Diego, CA, USA). Intensity data were analyzed by means of the Kruskal Wallis test with the Dunn's post hoc test. Results were considered significant at *P* < 0.05. There was no difference in intensity or shape for any of the bones between the solvent and medium controls. Rosiglitazone showed significant differences in bone intensities compared to the solvent control (see Figs. 8 and 9). The parasphenoid was less intensely stained, whereas the right utricular otolith and right circular otolith, the left and right entopterygoid, and the left and right branchiostegal rays 2 were more intensely stained. There was no effect on the shape of the bones. This skeletal staining will be further validated with proprietary and non-proprietary compounds in a consortium effort of the European Teratology Society.

## Declaration of Competing Interest

The authors declare that they have no known competing financial interests or personal relationships that could have appeared to influence the work reported in this paper.

## Appendix Supplementary materials

Image, application 1
