# Supplementary material for: Refinement of the zebrafish embryo developmental toxicity assay
Source: MethodsX. 2020 Oct 7;7:101087. doi: 10.1016/j.mex.2020.101087 (PMC7588703; doi:10.1016/j.mex.2020.101087)
Supplement: Supplementary file 1 [file mmc1.pdf]

Illustration of gross morphology endpoints in zebrafish embryos and larvae

(source: www.zebrafishlab.be)

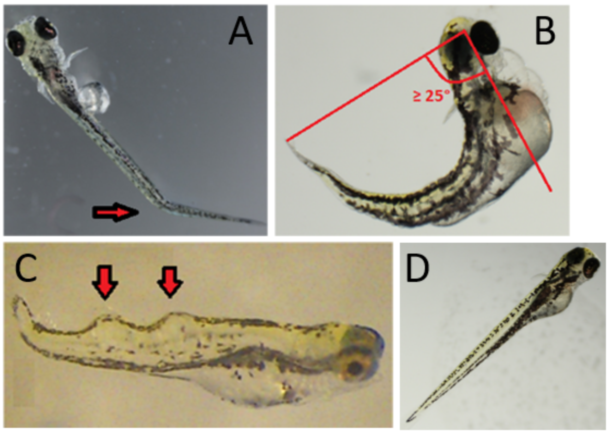

Supplementary Figure 1. Tail malformations in zebrafish larvae. A) Elbow, B) Curve (if curve ≥25°), C) Tissue deviation and D) No tail malformations.

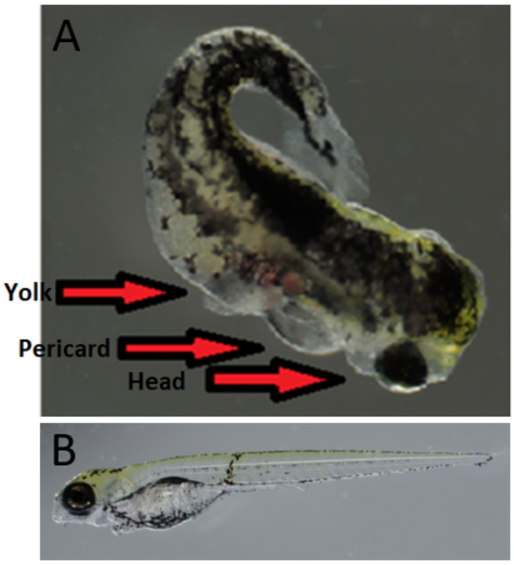

Supplementary Figure 2. Edema in zebrafish larvae. A) Yolk, pericard and head edema, B) No edema.

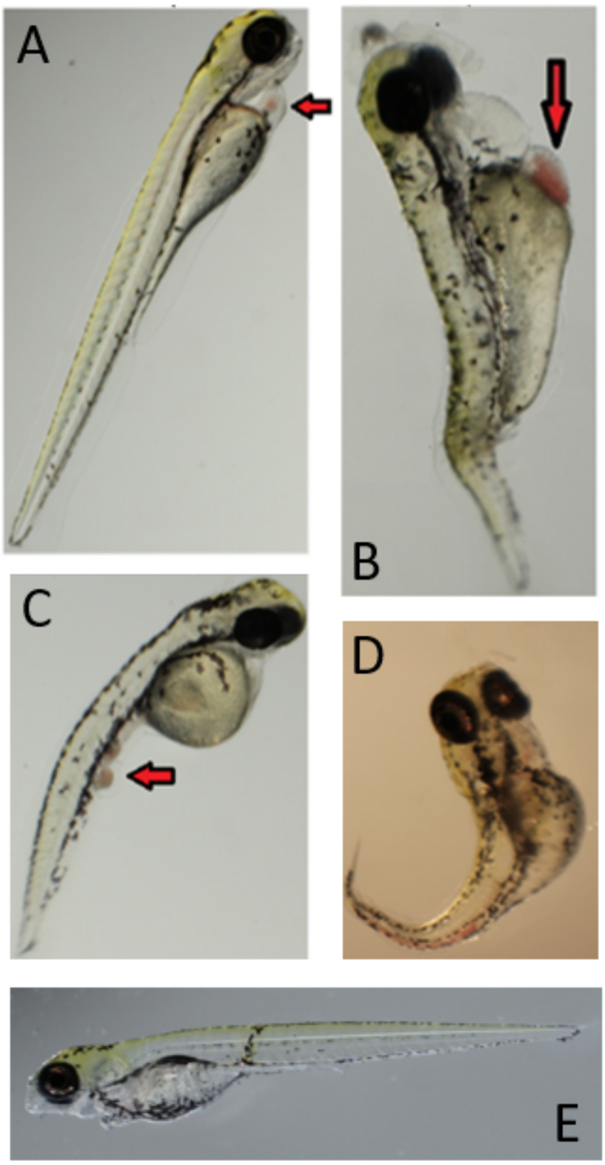

Supplementary Figure 3. Blood accumulation in zebrafish larvae in: A) the pericard, B) the yolk, C) the yolk extension and D) the tail. E) No blood accumulation.

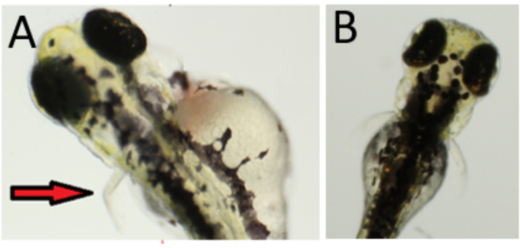

Supplementary Figure 4. Fin malformations in zebrafish larvae. A) Curved fin, B) Normal fins.

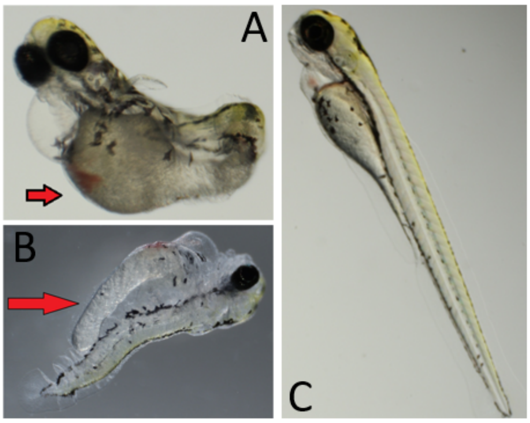

Supplementary Figure 5. Yolk malformations in zebrafish larvae. A&B) Larva with malformed yolk, C) No yolk malformations.

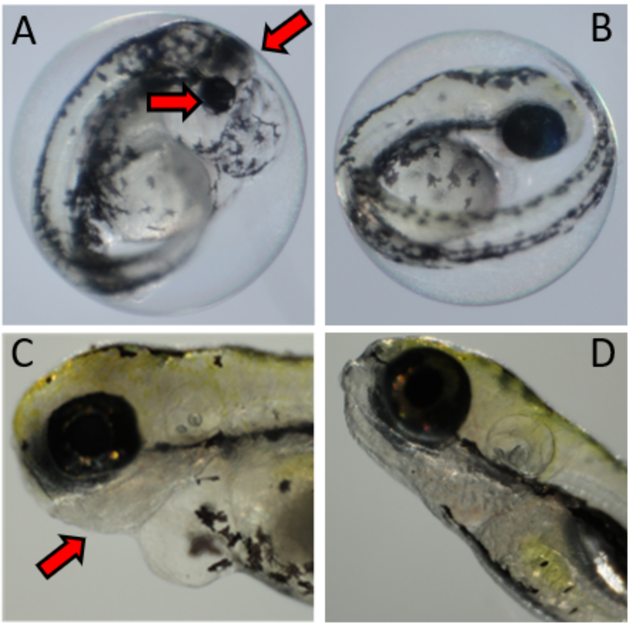

Supplementary Figure 6. Malformations of the head. A) Malformation of the eye and head shape, B&D) No head malformations, C) Malformation of the mouth.

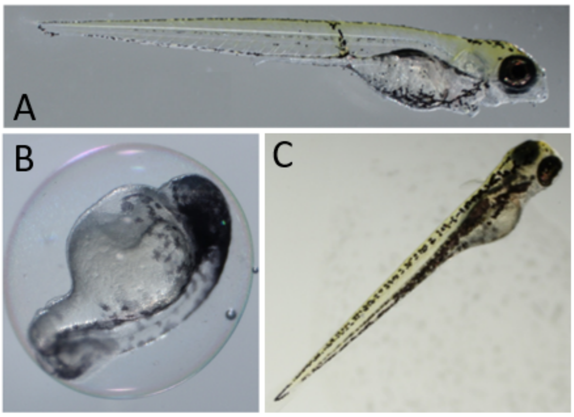

Supplementary Figure 7. Deviating pigmentation. A&B) Larva with deviating pigmentation, C) Normal pigmentation.

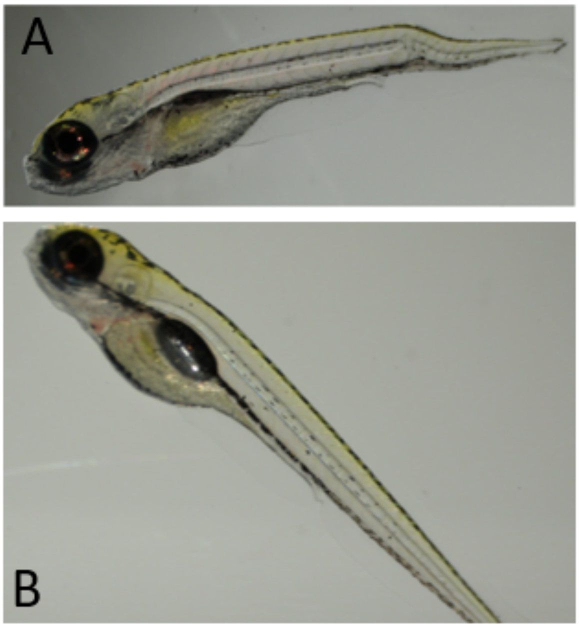

Supplementary Figure 8. Deviating swim bladder. A) Swim bladder not inflated. B) Swim bladder inflated, normal.
